# Emerging functions and clinical applications of exosomes in human oral diseases

**DOI:** 10.1186/s13578-020-00424-0

**Published:** 2020-05-24

**Authors:** Qiao Peng, Jing-ya Yang, Gang Zhou

**Affiliations:** 1 The State Key Laboratory Breeding Base of Basic Science of Stomatology (Hubei-MOST) and Key Laboratory of Oral Biomedicine Ministry of Education, School and Hospital of Stomatology, Wuhan University, Wuhan, China; 2 Department of Oral Medicine, School and Hospital of Stomatology, Wuhan University, Luoyu Road 237, Wuhan, China

**Keywords:** Exosomes, Oral disease, Oral squamous cell carcinoma, Primary Sjögren’s syndrome, Periodontitis, Oral tissue regeneration

## Abstract

Exosomes are cell-derived membranous vesicles of endosomal origin secreted by all type of cells and present in various body fluids. Exosomes are enriched in peptides, lipids, and nucleic acids, emerging as vital modulators in intercellular communication. Exosomes are increasingly being evaluated as biomarkers for diagnosis and prognosis of diseases, because the constituents of exosomes could be reprogrammed depending on the states of diseases. These features also make exosomes a research hotspot in oral diseases in recent years. In this review, we outlined the characteristics of exosomes, focused on the differential expressions and altered biological functions of exosomes in oral diseases, including oral squamous cell carcinoma, oral leukoplakia, periodontitis, primary Sjögren’s syndrome, oral lichen planus, as well as hand foot and mouth disease. Besides, accumulated evidence documents that it is implementable to consider the natural nanostructured exosomes as a new strategy for disease treatment. Herein, we highlighted the therapeutic potential of exosomes in oral tissue regeneration, oncotherapy, wound healing, and their superiority as therapeutic drug delivery vehicles.

## Background

**Fig. 1:**
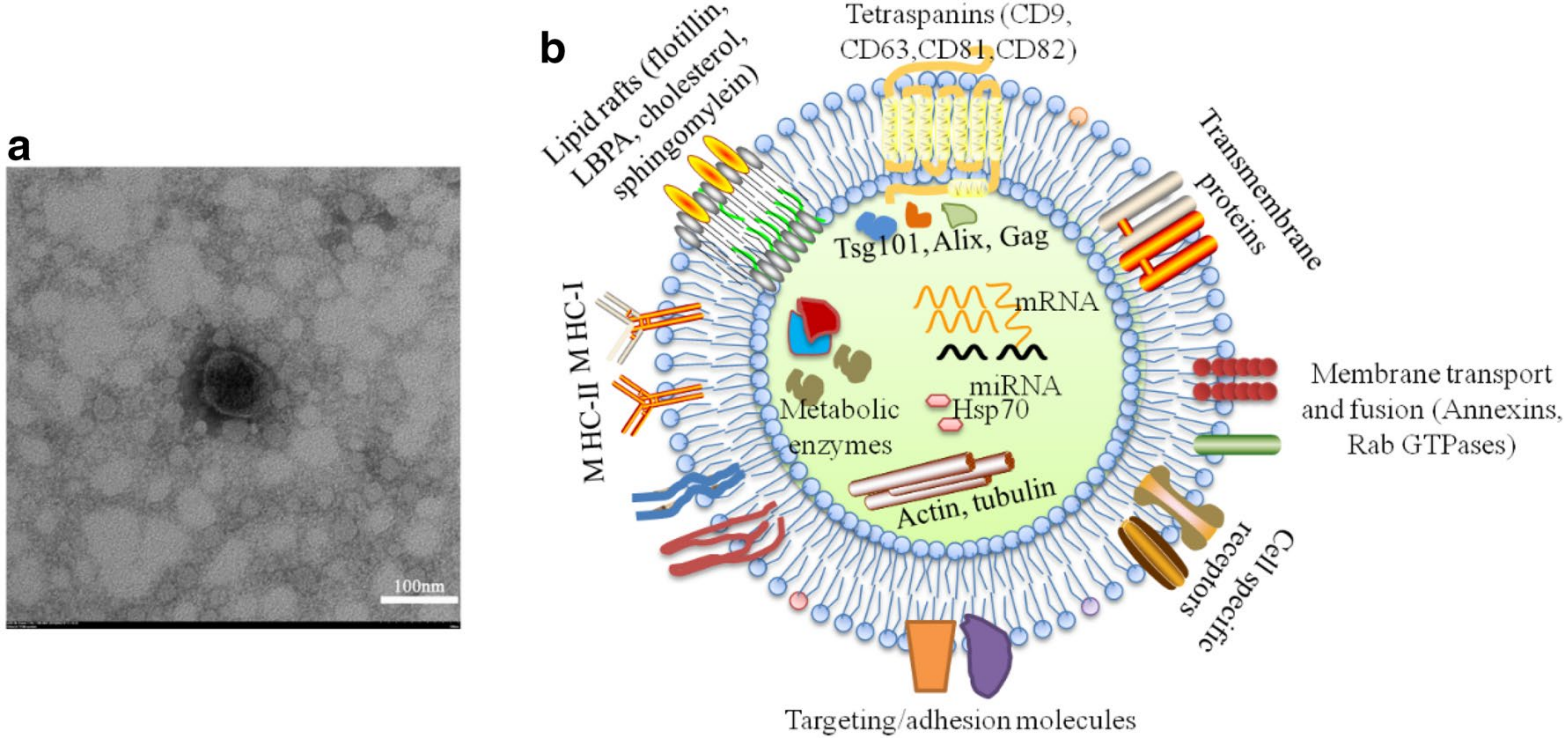
Characteristics of exosomes. **a** electron microscopic image of exosomes. Exosome showed a characteristic lipid bilayer with an average thickness of ∼ 5 nm and typical cup-shaped morphology, appearing as flattened spheres with diameters ranging from 30 to 100 nm. **b** Main constituent of molecules included in exosomes. Many proteins are common among all exosomes regardless of their maternal cell types, including tetraspanins, flotillin, heat shock proteins (HSP70, HSP90), MHC I, GTPases (Rab, RAL) and endosome-associated proteins (Alix, Tsg101). Exosomes also enrich in lipid rafts on the surface, including flotillin, LBPA, cholesterol, sphingomylein, and nucleic acids in the lumen, including DNAs (mtDNA, ssDNA, dsDNA), and RNAs (mRNA, miRNA, rRNA, and tRNA)

It has been more than 30 years since exosomes were first described as small vesicles which were generated during the process of reticulocyte maturation and mediated the selective externalization and removal of transferrin receptor from the erythrocyte [1]. Exosomes have a characteristic lipid bilayer with an average thickness of about 5 nm and a cup-shaped morphology, appearing as flattened spheres with diameters ranging from 30 to 150 nm [2] (Fig. 1a). Exosomes are derived from almost all types of cells and present in various biological fluids, such as plasma, serum, saliva, urine and human milk [1, 3–5]. In recent years, exosomes represent a new signaling paradigm to mediate intercellular communication because of their capacity to exchange components, including proteins, nucleic acids, and lipids [6, 7] (Fig. 1b).

The critical involvement of exosomes in different types of diseases may clarify the potential mechanisms of pathological processes. At present, tumor-derived exosomes are of most interest, because of their promotion in tumor proliferation, migration and invasion ability, and their contribution to immune suppression in tumor microenvironment [8, 9]. In addition, exosomes are reported to play a role in regulating inflammatory and immune diseases, such as rheumatoid arthritis, Sjogren’s syndrome and systemic lupus erythematosus [10]. It was reported that TNF-α^+^ exosomes promoted the T cell mediated pathogenesis of rheumatoid arthritis by inhibiting T cell-activation induced death [11]. Meanwhile, other studies focus on the potentially clinical applications of exosomes in tissue regeneration, targeted therapy, artificial exosome mimetics, or as biomarkers [12, 13]. For example, the combination of exosomes from human adipose stem cells and polydopamine-coating PLGA scaffold successfully accelerated the restoration of critical-sized mouse calvarial defects [14]. Zheng et al. found that proteasome subunit alpha type 7 (PSMA7) was remarkably higher in patients with inflammatory bowel disease (IBD) than healthy controls, which indicated that exosomal PSMA7 may be a biomarker for IBD diagnosis, therefore releasing patients from the pain of colonoscopy [15].

Recent studies have revealed the multifaceted roles of exosomes in oral diseases. Oral cancer-derived exosomes exacerbated the malignancy of cancers [16–19]. Li et al. proved the hypoxic oral squamous cell carcinoma (OSCC) cells secreted miR-21-rich exosomes in a HIF-dependent manner [20]. Increased exosomal miR-21 markedly enhanced the expression of snail and vimentin, but decreased E-cadherin level in OSCC cells, which ultimately contributed to the migration and invasion of OSCC cells [20]. Exosomes were also a kind of message transmitter that transmitted signals between tumor cells and other type cells. Exosomal miR-29a-3p from OSCC cells promoted M2-type macrophages polarization, and such macrophages enhanced the proliferation and migration of OSCC cells [21]. The ubiquitous existence of exosomes in human body fluids makes exosomal composition promising biomarkers for real-time monitoring in clinical application. In our previous work, circulating exosomal miRNAs were identified differentially expressed in oral lichen planus (OLP) patients. Especially, the increased expression of circulating exosomal miR-34a-5p in OLP was positively correlated with the disease severity [22]. Of importance, in regenerative medicine, exosomes derived from oral mesenchymal stem cells (MSCs) were able to regenerate oral tissues such as dental pulp and periodontal tissues [23–26].

Based on the current knowledge, we describe the mechanisms of exosomes formation and signal transmission, and summarize the latest studies on the roles of exosomes in different oral diseases. Moreover, we emphasize the potentially clinical applications of exosomes on oral tissue regeneration, oncotherapy, wound healing, and as therapeutic drug vehicles for oral diseases.

## Characterization of exosomes

Exosomes originate from an endocytic compartment. Originally, early endosome is formed by inward budding of plasma membrane. During maturation of early endosome, the inward budding of limited areas of the endosomal membrane to form intraluminal vesicles (ILVs) produces multivesicular bodies (MVBs), also known as late endosomes [27]. During the inwarding process of ILVs, many cytoplasmic components are encapsulated. Two fates have been identified for MVBs: some of them deliver to lysosomes or autophasome for degradation, while others fuse with the plasma membrane. In the former process, MVBs directly fuse with lysosomes to form autolysosome or fuse with autophasosome to form amphisome, where endocytosed cargos are degraded; while, in the latter process, MVBs fuse with plasma membrane inducing exosome secretion [28, 29]. It is usually considered that exosome secretion requires formation of an endosomal sorting complex required for transport (ESCRT) machinery [22].

**Fig. 2:**
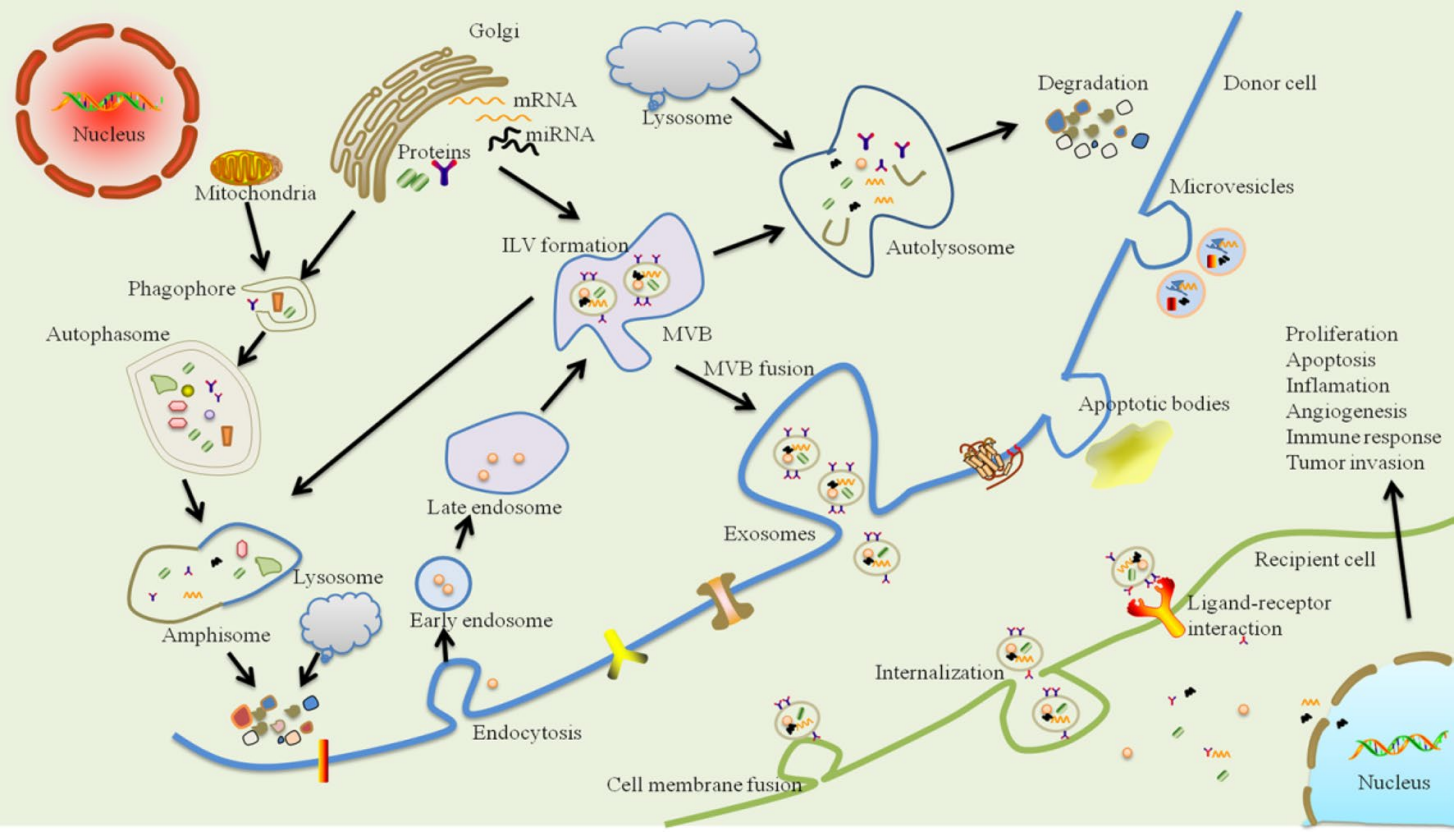
Schematic representation of exosome biogenesis, release and intercellular communication. Exosomes originate from an endocytic compartment. Early endosome is formed by the inward budding of plasma membrane. During maturation of early endosome, the inward budding of limited areas of the endosomal membrane to form intraluminal vesicles (ILVs) produces multivesicular bodies (MVBs). MVBs faced two fates, where some of them are delivered to lysosomes or autophasome for degradation, while others fuse with the plasma membrane inducing the secretion of exosomes. During the inwarding process of ILVs, many cytoplasmic components are encapsulated, such as proteins, lipids and nucleic materials, which makes them represent a new signaling paradigm to interfere cell-to-cell communication. Moreover, this intercellular signal transmission might be mediated through three pathways, including endocytosis/internalizatioin, direct membrane fusion, or receptor-ligand interaction

Evidence demonstrated that exosomes were closely associated with cell proliferation, apoptosis, antigen presentation, immune regulation, tissue regeneration and tumor initiation [30–32]. How exosomes transmit the signals into incorporating cells is still an unresolved question, but three potential mechanisms have been demonstrated (Fig. 2): (1) exosomes are endocytosed/internalized and then fuse with the endosomal limiting membrane of recipient cells; (2) exosomal membranes directly fuse with the plasma membrane of recipient cell; (3) exosomes attach to recipient cell surface by receptor-ligand interaction [33, 34]. After reception, it is the diverse biological composition within the exosomes, including proteins, lipids and nucleic acid that functions in exosomes mediate intercellular signaling transmission, which play key roles in maintaining homeostasis and regulating the physiopathological processes [35, 36].

## Exosomes in oral diseases

### Exosomes in oral squamous cell carcinoma

Oral squamous cell carcinoma (OSCC) is the most common epithelial cancer of the head and neck [37, 38]. OSCC is highly malignant and prone to local invasion and cervical lymph node metastasis, causing facial deformity, speech and esthetic disorders and leading to low survival rate and poor quality of life [38].

### Proteins in OSCC cell-derived exosomes

Exosomal proteins can promote tumor development in both paracrine and autocrine ways. Exosomes containing EGFR from OSCC cells were capable of transforming normal epithelial cells into a mesenchymal phenotype in a paracrine fashion [17]. Hou et al. found that exosomes from salivary adenoid cystic carcinoma cell line SACC-83 enhanced the migration and invasion ability of the parental SACC-83 by targeting cell junction-associated proteins, including claudin-1, ZO-1, and β-catenin [16, 39].

The protein composition in OSCC cell-derived exosomes (OSCC-Exos) could be changed significantly after ionizing radiation [40, 41]. This alteration reinforced the exosome secretion, the survival of irradiated OSCC cells, and the proliferation of non-irradiated OSCC cells, because exosomes were able to repair the broken DNA-double strand [40]. Furthermore, differential expression patterns of protein in OSCC-Exos probably reflected the states of disease. Ono et al. revealed that exosomes secreted by metastatic phenotype of OSCC cells comprised larger amount of oncogenic proteins, including EpCAM, EGFR, and HSP90 than their parental OSCC cells. In addition, highly expressed HSP90, TRAP1 and HSP105 were correlated with poor prognosis of OSCC and thus could be potential prognostic biomarkers for OSCC [42].

Moreover, OSCC-Exos internalized by NK cells could up-regulated the expression of interferon regulatory factor 3 (IRF-3) and its phosphorylation by releasing NF-κB-activating kinase-associated protein 1 into NK cells [43]. Overexpressed IRF-3 drove NK cells to express type I interferon, chemokine and costimulatory molecules, hence enhancing their tumor-suppressing functions, including cell proliferation, release of perforin and granzyme M, and cytotoxicity toward tumor cells [43].

### MiRNAs in OSCC cell-derived exosomes

Hypoxia is a common feature of OSCC and associated with aggressiveness and poor outcomes [44]. Li et al. found that exosomes purified from supernatants of hypoxic OSCC cell lines SCC-9 and CAL-27 significantly overexpressed miR-21 in a HIF-1a and HIF-2a–dependent manner, which promoted the migration and invasion of OSCC cells in vitro and induced tumor growth and metastasis in xenograft mice model [20]. Interestingly, exosomes derived from cisplatin-resistant OSCC cells could transfer miR-21 into their OSCC parental cells [18]. This transference of miR-21 exerted an enhancement effect on chemoresistance and decreased the DNA damage signaling in response to cisplatin by targeting phosphatase and tensin homolog and programmed cell death 4 [18]. Therefore, hypoxia and cisplatin treatment may simultaneously stimulate tumor cells to generate miR-21-rich exosomes, which in turn reinforce the prometastatic behaviors and the resistance to chemotherapy, respectively.

Sakha et al. identified that exosomes secreted from highly metastatic human oral cancer cell line HOC313-LM (HOC313-LM-Exo) could induce cell growth via activating the ERK and AKT pathways. HOC313-LM-Exo expressed highly amount of miR-342–3p and miR-1246, enhancing the cell motility of its parental cell line HOC313 and the establishment of the metastatic niche by communication between cancer cells and normal cells. Exosomal miR-1246, in particular, was significantly associated with the malignancy by directly targeting DENN/MADD Domain Containing 2D [19].

OSCC cell-derived exosomal miRNAs can exacerbate the severity of disease not only by functioning on the OSCC cells itself, but also by boosting the M2-polarization of macrophages. Macrophages are documented to play critical roles in the tumor microenvironment. With the induction of tumor cells they can differentiate into tumor-associated macrophages (TAM), which is similar to M2-like phenotype polarization, showing great diversity and plasticity [45]. We have previously identified that the expression of CD163^+^ macrophages (M2 macrophages) was higher in oral leukoplakia (OLK) and OSCC than that in normal oral mucosa [46, 47]. Others also proved a marked positive correlation between the increased CD163^+^ macrophages and the pathological grade of OSCC [48], supporting the importance of M2 macrophages in the progression of OLK and OSCC. Recently, a report found the positive participation of OSCC-Exos in M2-subtype macrophages polarization [21]. This M2 polarization was induced by highly-expressed miR-29a-3p in OSCC-Exos, which negatively regulated the activity of SOCS1/STAT6 signaling pathway, aggravating the proliferation and invasion of SCC9 and CAL-27 [21].

### Carcinoma associated-fibroblast-derived exosomes

Carcinoma associated-fibroblasts (CAFs) are a highly enriched cellular stromal component of many solid tumors [49, 50]. By secreting diverse cytokines, growth factors and chemokines, CAFs enhanced the drug-resistance acquisition, induced the EMT, and contributed to the progression, invasion, metastasis, and angiogenesis of cancer cells [51]. Recently, extracellular vesicles were reported to play a central role in the crosstalk between tumor cells and CAFs [52–55].

Jiang et al. showed that normal human gingival fibroblasts exhibited a phenotype switch to CAFs after co-culture with CAL-27-derived microvesicles [55]. The microvesicle-activated CAFs in turn promoted the migration and invasion of OSCC cells via producing more lactate [55]. In addition, exosomal miR-155 derived from melanoma cells upregulated the expression of proangiogenic factors, such as VEGF, FGF2, MMP9, in CAFs by targeting SOCS1/JAK2/STAT3 signaling pathway, which resulted in the increase of melanoma angiogenesis [56].

On the other hand, CAF-derived exosomes modified the metabolic reprogramming of cancer cells, and upregulated the expression of invasion-associated genes, such as ROCK2, FLOT1 and FAM129B [54]. Li et al. found that the expression of miR-34a-5p in CAF-derived exosomes was significantly reduced, while overexpression of exosomal miR-34a-5p could inhibit the tumorigenesis of OSCC [53]. After binding with its direct downstream target AXL, miR-34a-5p strengthened OSCC malignancy through directly targeting the AKT/GSK-3β/β-catenin/Snail signaling pathway [53]. Besides, Languino et al. proved that, in OSCC, CAF-derived exosomes could stimulate the TGF-β signaling pathway in keratinocytes by exosomal TβRII [52], which enhanced the possibility that TGF-β signaling might be influenced by intercellular communication between tumor and the microenvironment.

### Body fluids-derived exosomes in OSCC

Liquid biopsy has been extensively investigated in recent years because of obvious advantages, such as its minimal invasiveness, painlessness, inexpensiveness, and repeatability [57]. Since biological cargos of exosomes from various physiological fluids changed greatly depending on different stages and types of diseases, it is of great potential to exploit exosomal cargos as biomarkers in the field of liquid biopsy.

### Salivary exosomes in OSCC

Zlotogorski-Hurvitz et al. identified that the size and concentration of OSCC-Exos from oral fluid were larger and higher than that of healthy individuals and the expression of CD81 was significantly lower in OSCC [58]. Gai et al. revealed an upregulation of miR-412-3p, miR-512-3p, miR-27a-3p, miR-373-3p and miR-494-3p in salivary exosomes from OSCC patients [59]; furthermore, miR-302b-3p and miR-517b-3p were expressed specifically only in samples from OSCC group [59].

In addition, proteome analysis showed that salivary exosomes isolated from OSCC patients were enriched in proteins related to the inflammatory system, transport of metals, as well as cellular growth and proliferation [60]. These functional biomolecules within the salivary exosomes were able to induce inflammatory cells migrate to the tumor sites through chemotactic mechanisms, which may be important in the subsequent immunoediting of inflammatory cells [60].

Fourier-transform infrared (FTIR)-based spectrum of salivary exosomes could differentiate OSCC from healthy individuals with a sensitivity of 100% and specificity of 89%, displaying a specific mid-infrared spectral signature for OC salivary exosomes [61]. This difference was caused by the subtle changes of exosomal proteins, lipids and nucleic acids in salivary from patients with OSCC [61].

### Circulating exosomes in OSCC

It was reported that plasma exosomes from OSCC exerted suppressive effects on immune system by downregulating the expression of NKG2D in NK cells [62]. In patients with active disease, plasma exosomes were more effective to establish an immune suppressive microenvironment by increasing the apoptosis of CD8^+^ T cells, inhibiting the proliferation of CD4^+^ T cells, and promoting the production of Treg cells [62]. These findings indicated that circulating exosomes from OSCC may contribute to the development of OSCC by suppressing the anti-cancer effects of NK cells and T cells.

In addition to affecting the anti-cancer immunity, circulating exosomes also displayed a tight connection with OSCC status. Compared to plasma free miRNAs, the expression profiles of plasma exosomal miRNAs from tongue SCC patients more resembled the tumor tissues [63]. Similarly, the level of PD-L1 carried by plasma exosomes instead of soluble PD-L1 level was correlated with disease severity, the UICC stage and the lymph node status [64]. Moreover, Wang et al. have identified a higher expression of laminin-332 in plasma exosomes from OSCC patients with lymph node metastasis [65]. Li et al. found that serum exosomal miR-21 level was closely associated with HIF-1a/HIF-2a expression, T stage, and lymph node metastasis [20]. Therefore, cargos in circulating exosomes were of potentiality to be used as novel diagnostic biomarkers for the surveillance of tumor conditions and lymph node metastasis in OSCC.

### Exosomes in oral leukoplakia

Oral leukoplakia (OLK) refers to a white patch or plaque of the oral mucosa that cannot be defined as a known disease or disorder and carries an increased risk of progressing to OSCC [66]. OLK is one of the most common oral premalignant disorders (OPMD) with malignant transformation rates of 2% to 5% [67].

A newly published study reported that exosomal miR-8485 secreted by MSCs derived from OLK with dysplasia played a promoting role in the proliferation, migration and invasion of DOK and SCC-15 cell lines [68]. Another research demonstrated that in hamster OPMD model, bone marrow-MSCs-derived extracellular vesicles with genetically modified overexpression of miR-185 (MSC-EV-miR-185) were capable of remarkably attenuating inflammation severity and decreasing degree of dysplasia in the OPMD tissue [69].The MSC-EV-miR-185 treatment obviously reduced the expression of proliferation marker PCNA and angiogenic marker CD31, and induced cell apoptosis in the buccal lesions, indicating their potential value as a novel therapeutic option for OPMD [69].

### Exosomes in periodontitis

Periodontitis is a chronic multifactorial inflammatory disease of supporting tooth structures initiated by dysbiotic plaque biofilms [70, 71]. It is primarily characterized by the loss of periodontal tissue support including clinical attachment loss, alveolar bone destruction, presence of periodontal pocketing and gingival bleeding, irreversibly impairing the integrity of the periodontium and finally leading to tooth loss [72, 73].

Periodontal ligament fibroblasts (PDLFs) are the main cell populations that contact pathogenic microorganisms in early periodontal inflammation [74]. After lipopolysaccharide (LPS) stimuli, human PDLF-derived exosomes slightly upregulated the expression of IL-6 and TNF-α in osteoblasts, and concomitantly significantly inhibited the expression of osteogenesis-related elememts, including collagen-I and osteoprotegerin, and reduced the activity of alkaline phosphatase [75]. PDLFs also engage in the maintenance of periodontal tissue homeostasis in the oral mechanical environment [76]. Stimulated with cyclic stretch, PDLFs secreted exosomes that could suppress IL-1β production in LPS-treated macrophages through the inhibition of NF-κB signaling pathway [77].

Periodontal ligament stem cells (PDLSCs) is a unique MSC population that displays self-renewal ability and multipotency when interacts with their surrounding inflammatory microenvironment [78]. Compared with exosomes extracted from normal PDLSCs, exosomes derived from LPS-stimulated PDLSCs contained a higher amount of miR‐155 and its downstream target Sirtuin‐1, which reduced the expression of Th17 but increased the expression of Treg, thereby alleviating the inflammation through the Th17/Treg/miR‐155‐5p/Sirtuin‐1 regulatory network [79].

In salivary, the level of exosomal PD-L1 mRNA was higher in periodontitis than controls, and high expressions of PD-L1 were associated with advanced stages of periodontitis [80]. On the contrary, the level of salivary CD9 and CD81 exosomes was reduced in periodontitis and negatively correlated with disease status [79, 81].

### Exosomes in primary Sjögren’s syndrome

Primary Sjögren’s syndrome (pSS) is a chronic autoimmune disorder characterized by focal lymphocytic infiltration of the exocrine glands, such as salivary gland and lacrimal gland, mostly leading to dry eyes and dry mouth [82, 83]. Although the exact etiology and pathogens still remain unclear, evidence imply the critical role of exosomes in the dysregulation of immune system in pSS.

In pSS, salivary gland epithelial cells (SGECs), one main source to secrete autoantigens such as Ro/SSA and La/SSB, played a pivotal role in the initiation and progression of pSS in the local immune response [84, 85]. Recently, SGECs was reported to secreted exosomes that were highly contained autoantigens of Ro/SSA, La/SSB and Sm, demonstrating a novel mechanism of autoantigen presentation causing the autoimmune response [86]. In addition, Aqrawi et al. identified novel potential biomarkers of APMAP, GNA13, WDR1 in saliva-derived exosomes and APEX1, PRDX3, CPNE1 in tear-derived exosomes from pSS that may be used as an additional diagnostic process to increase diagnostic accuracy [87].

Epstein–Barr virus (EBV) was considered as another important factor contributing to the pathogenesis of pSS because of its tropism for salivary glands and the ability to preferentially infect B cells [88]. Gallo et al. showed that exosomal ebv-miR-BART13-3p derived from EBV-infected B cells was functionally transferred into SGECs [89]. The exosomal ebv-miR-BART13-3p directly targeted stromal interacting molecule 1 (STM1) in SGECs, and then resulted in loss of store operated Ca2^+^ entry and Ca2^+^-dependent activation of nuclear factor of activated T cells, leading to the salivary dysfunction in pSS [89].

## Exosomes in oral lichen planus

Oral lichen planus (OLP) is a common inflammatory autoimmune disease involving oral mucosa with unclarified etiology [90]. Clinically, OLP usually presents as symmetrical, bilateral or multiple lesions with 6 different clinic patterns of reticular, papular, plaque, erosive, bullous, and atrophic [91]. Pathogenesis of OLP has not been completely elucidated yet. However, T cell-mediated antigen-specific mechanism was found to play a key role in the pathogenesis of OLP, which leaded to the disruption of basement membrane by inducing the apoptosis of keratinocytes [92].

Recent studies indicated that the aberrant expression of exosomal miRNAs might participate in the development of OLP. J-S Byun et al. reported that exosomal miR-4484 from salivary was increased in patients with OLP [93]. Our group showed that plasma-derived exosomal miR-34a-5p and miR-130b-3p were significantly upregulated while exosomal miR-301b-3p was downregulated in OLP, and a positive correlation was found between expression of exosomal miR-34a-5p and the severity of OLP [22]. We also found that circulating exosomes from OLP, especially the erosive type, could significantly enhance T cell proliferation and attenuate the apoptosis, and remarkably increase the migration capacity of T cells as well as the ratio of IFN-γ/IL-4, potentially accelerating the OLP progression by regulating the T cell-mediated inflammatory response [94].

## Exosomes in hand, foot and mouth disease

Hand, foot, and mouth disease (HFMD) is a worldwide epidemic acute viral illness, in which two major causative agents human enterovirus 71 (EV71) and coxsackievirus A16 (CVA16) account for more than 70% of cases in recent outbreaks [95, 96]. The extremely severe HFMD (ESHFMD) mainly caused by EV71 has severe neurologic clinical symptoms and significant fatalities [97].

Jia et al. validated three significantly differential expressed serum exosomal miRNAs (miR-671-5p, miR-16-5p and miR-150-5p) between children with HFMD and healthy controls, where exosomal miR-671-5p and miR-150-5p were decreased while exosomal miR-16-5p was increased in patients with a specificity of 72–100% and sensitivity of 78–100% [98]. In addition, significant difference was also identified among the three exosomal miRNAs between mild HFMD and ESHFMD, where miR-671-5p was only detectable in healthy and mild HFMD, providing supplemental biomarkers for subtyping HFMD infections [98] (Table 1).

**Table 1:** miRNAs expression patterns in human oral diseases

Diseases	Source	Exosomal miRNAs	Amounts	Effects of exosomal miRNAs	References
Oral squamous cell carcinoma	Hypoxic SCC-9 and CAL-27	miR-21	Up	CAL-27-derived exosomes was maily used in functinal experiments.Exosomal miR-21 induced prometastatic behaviors under hypoxia condition	[20]
	cisplatin-resistant HSC-3 and SCC-9	miR-21	Up	exosomes transferred miR-21 to OSCC parental cells and induced cisplatin resistance	[18]
	HOC313-LM	miR-1246, miR-342–3p	Up	Increased the migration and invasion ability of HOC313-P cells. exosomal miR-1246 enhanced cell motility	[19]
	SCC-9, CAL-27	*miR*-*29a*-*3p*	up	promoted M2-subtype macrophages polarization via SOCS1/STAT6 signaling pathway	[21]
	Primary cancer-associated fibroblasts	miR-34a-5p	Down	miR-34a-5p/AXL axis promoted OSCC progression, inducing the EMT to promote cancer cells metastasis	[53]
	Saliva	miR-412-3p, miR-512-3p, miR-27a-3p, miR-373-3p and miR-494-3p, miR-302b-3p, miR-517b-3p	Up	Potentially be used in liquid biopsy; miR-302b-3p, miR-517b-3p expressed specifically only in samples from OSCC saliva	[59]
	Serum	miR-21		Closely associated with HIF-1a/HIF-2a expression, T stage, and lymph node metastasis	[20]
	Plasma	hsa-miR-19a, hsa-miR-512-3p, hsa-miR-27b, hsa-miR-20a, hsa-miR-28-3p, hsa-miR-200c, hsa-miR-223, hsa-miR-20b, hsa-miR-151-3p	Up	More reliable for evaluation of circulating tumor-miRNA expression than plasma	[63]
		hsa-miR-22, hsa-miR-370, hsa-miR-139-5p, hsa-let-7e, hsa-miR-145-3p, hsa-miR-30c	Down		
Primary Sjögren’s syndrome	EBV-infected B cells	ebv-miR-BART13-3p	Not shown	Decreased store operated Ca2^+^ entry and Ca2^+^-dependent activation of NFAT in SGECs	[89]
Oral lichen planus	Saliva	miR-4484	Up	Potential miRNA biomarker for OLP	[93]
	Plasma	miR-34a-5p, miR-130b-3p	Up	miR-34a-5p was positively correlated with the severity of OLP	[22]
		miR-301b-3p	Down	Not shown	
Hand, foot and mouth disease	Serum	miR-16-5p	Up	Biomarkers for diagnose, and miR-671-5p may be used for subtyping HFMD	[98]
		miR-671-5p, miR-150-5p	Down		
Oral leukoplakia	MSCs from human primary tissues	miR-8485	Up	Promoted the proliferation, migration and invasion of DOK and SCC-15 cell lines	[68]
	Murine bone marrow-MSCs	miR-185	Up*	Attenuated inflammation severity and decreased degree of dysplasia in the OPMD tissue	[69]
Periodontitis	LPS-stimulated periodontal ligament stem cells	miR‐155	Up	Reducing the expression of Th17 and increasing the expression of Treg	[79]

Up: the expression of miRNAs was upregulated in exosomes

Up*: the the expression of miRNAs in exosomes was upregulated by transfection

Down: the expression of miRNAs was down regulated in exosomes

## Present and future prospects of exosomes on oral treatments

### Therapeutic potential of mesenchymal stem cell-derived exosomes

Mesenchymal stem cells (MSCs) are progenitor cells with differential potential and self-renewable capacity [99], originally isolated from bone marrow and subsequently from other tissues [100]. In addition to the well-known bone marrow-derived MSCs (BM-MSCs) and adipose-derived stem cells (ADSCs), in the field of dental research, several types of dental stem cells isolated from mature and immature teeth, such as dental pulp stem cells (DP-MSCs), stem cells derived from dental pulp of human exfoliated deciduous teeth (SHEDs) and periodontal ligament stem cells (PDLSCs), are attracting more and more attention [99].

Notably, in the latest studies, MSCs-derived exosomes are increasingly recognized as promising strategies to alleviate tissue injury and promote tissue regeneration in dental treatment, including dental pulp regeneration, oral oncotherapy and periodontal regeneration.

### Exosomes and dental pulp regeneration

Huang et al. showed that exosomes derived from DP-MSCs (DP-MSCs-Exos) cultured under odontogenic differentiation conditions triggered dental pulp-like tissue regeneration, such as increased expression of DMP1, DPP and active blood vessels in a tooth root-slice model [101]. Normally, dental pulp is highly vascularized which is fundamental to nutrient supply, waste removal and anti-inflammatory response [102]. It was reported that dental pulp cells-derived exosomes contributed to the vascularization via promoting the proliferation, pro-angiogenic factor expression (VEGF-A, MMP-9, FGF-2, KDR), and tube formation of human umbilical vein endothelial cells [103].

In addition, the tooth development was modulated by interaction between the epithelial cells and mesenchymal cells, and exosomes was critical in this process. Exosomes derived from epithelial cells promote mesenchymal cells to produce dentin sialoprotein and undergo mineralization while exosomes secreted by mesenchymal cells escalated the expression of basement membrane components, ameloblastin and amelogenenin in epithelial cells [104]. Moreover, it is worth noting that schwann cells, the principal glial cells in peripheral nervous system, played an important immunomodulatory role in dentin repair. Exosomes from schwann cells accelerated the proliferation and matained the multipotency and self-renewal capacities of dental pulp cells through upregulation of Oct4, Sox2 and Nanog, providing great application potential in tissue regeneration [105].

To be brief, exosomes may lead to dental pulp regeneration via increasing the expression of specific proteins, promoting the vascularization, modulating the interaction between epithelial and mesenchymal cells, and augmenting the abilities of dental pulp cells, which might serve as an important therapeutic method in the future.

### Exosomes and oncotherapy

In 2016, Altanerova et al. demonstrated that DP-MSC-Exos may inhibit tumor through its dual tumor cell killing activity. For one thing, exosomes released from DP-MSCs that transduced with yCD::UPRT mRNA (yCD::UPRT-DP-MSCs-Exos), a suicide gene, could be internalized by tumor cells and allow tumor cells to translate nontoxic 5-FC to toxic 5-FU, leading to cancer cell death in a dose-dependent manner in the presence of a prodrug 5-fluorocytosine [24]. For another, the yCD::UPRT-DP-MSCs-Exos labeled with iron oxide (Venofer) behaved as magnetic nanoparticles, which were more effective at inducing tumor cell death by alternating magnetic field-induced intracellular hyperthermia and/or by exposure to the prodrug 5-FC [24, 67]. Moreover, exosomes derived from menstrual mesenchymal stem cells (MenSC-Exos) exerted an antitumor effect by decreasing the angiogenesis in OSCC model [106].

Besides, exosomes derived from SHEDs (SHEDs-Exos) displayed potent anti-inflammatory properties by suppressing edema, cathepsin B and MMPs induced by carrageenan in mice [107]. Under oxidative stress conditions, SHEDs-Exos was reported to show neuroprotective abilities via inhibiting the apoptosis of dopaminergic neurons [108].

### Exosomes and periodontal regeneration

It was found that MSCs mediated periodontal regeneration through secretion of exosomes. In rats with periodontal intrabony deficiencies, collagen sponges that contained human MSCs-Exos could more efficiently repair the defects than control rats with newly-formed bone and PDL, and concomitantly lead to the proliferation of PCNA^+^ cells [25]. The followed experiments performed in PDL cells proved that, human MSCs-Exos could be rapidly taken up by PDL cells and promoted the migration and proliferation of PDL cells through CD73-mediated adenosine receptor activation of AKT and ERK signaling pathways [25]. Researchers also observed that exosomes secreted by adipose-derived stem cells (ASCs-Exos), exerted a better therapeutic effect on ligature-induced periodontitis compared to ADSCs themselves, which manifested as a higher area of newly formed tissues [109].

### Wound healing efficiency of exosomes

The oral mucosa displays unique regenerative properties such as foetal-like wound healing and antibacterial properties [110, 111]. On contrary to the “reparation” of skin wounds that heal with scar, the “regeneration” of wounds in the oral mucosa tends to heal without scar [112]. Therefore, sheets of oral mucosal epithelial cells (OMECs) are currently used after endoscopic removal of superficial tumours in the esophagus to alleviate esophageal stricture and improve mucosal healing.

The latest research showed that, exosomes isolated from conditioned media of human OMECs sheets exhibited pro-regenerative effects on skin wound healing [26]. These exosomes attenuated the proliferation of human skin fibroblasts in a dose-dependent manner and considerably increase the gene expression of growth factor HGF, VEGFA, FGF2 and CTGF in vivo, and significantly reduced the wound size in rat models in vitro [26]. These findings revealed the clinical application potential of combining cell sheets with exosomes in the future treatment of patients with early esophageal cancer.

## Application of exosomes as therapeutic drug delivery vehicles

Although chemotherapy have already exhibited excellent therapeutic effects on OSCC treatment at present, it often shows severe side effects. In recent years, nanodrug delivery system have emerged as a key advance in cancer treatment for its multiple advantages than traditional drugs, including diminished drug degradation, increased targeting efficiency and prolonged drug release [113]. Notably, exosomes derived from different cells with specific cell tropism and enhanced ability to target specific tissues or organs are newly recognized natural nanocarriers [114, 115]. Besides, it seems more easier for exosomes to escape phagocytosis by the mononuclear phagocyte system than the synthetic nanodrugs, making them function as an “invisibility cloak” for incorporated therapeutic agents [116].

Signal regulatory protein α (SIRPα), a cell-surface protein mainly expressed on macrophages and dendritic cells, could bind to CD47 [117]. In our previous study, we identified that CD47 was overexpressed in OSCC lesions and cell lines, and the CD47-SIRP-α interaction inhibited the engulfment of tumor cells by macrophages and promoted M2 macrophages differentiation, mediating the anti-phagocytosis and immune escape of OSCC cells [46, 47]. Accordingly, Koh et al. established a type of exosomes engineered with SIRPα variants (SIRPα-exosomes) [118]. The SIRPα-exosomes significantly increased the phagocytic capacity of macrophage, attenuated tumor growth. Moreover, the SIRPα-exosome based platform remarkably augmented T cell infiltration in syngeneic mouse models of cancer [118]. It is plausible to speculate that exosomes equipped with specific antagonists, an emerging strategy for nano-immuno cancer therapy, may be promising for tumor treatment in the future.

Paclitaxel, performing best to induce the apoptosis of OSCC cells (40–50%) in comparison with daunarubicin, doxorubicin and vincristine, and exhibiting a highest negative correlation with multiple drug resistance (MDR)-linked gene expression, may be the best choice of treatment for the studied OSCC patients [119]. Kim et al. showed that the incorporation of paclitaxel into exosomes significantly augmented paclitaxel accumulation in drug-resistant lung cancer cells and was 50 times more cytotoxic than conventional paclitaxel in vitro [116]. All the results imply the superior inhibition of exosomes carrying paclitaxel on OSCC cells and merit further exploration and confirmation.

Efforts to develop RNA interference therapeutic technology have been significantly intensified for biomedical application. Delivering short interfering RNA (siRNA) to recipient cells is an effective method to selectively suppress target mRNA of interest, showing great potential for use in disease treatment [120]. However, naked siRNA is rapidly degraded by nucleases in the blood circulation and might fail to pass into target cell membranes due to their negatively charged surface [121, 122]. Encapsulation of siRNAs in exosomes is a promising novel strategy to overcome most of these delivery issues. It is noteworthy that DNA damage-related response plays a central role in maintaining genomic stability and cellular survival. RAD51, as a DNA repair gene, is involved in DNA damage response, cell cycle checkpoint and maintains the stability of the gene [123, 124]. Overexpression of RAD51 was documented in diverse human tumors, and was predicted significantly related to OSCC prognosis [125]. Shtam et al. have successfully transfected two different siRNAs against RAD51 and RAD52 into exosomes [121]. They proved that exosome-delivered RAD51 siRNA functionally inhibited RAD51 expression in tumor cells, induced the accumulation of cells in S and G2/M phases, and then caused massive reproductive cell death [121]. Similarly, encapsulation of RAD51 siRNA into exosome is of great potential to improve OSCC therapeutic efficiency.

## Conclusions

Growing evidence suggests that exosomes act as an important regulator in oral diseases. Exosomes derived from OSCC cells and body fluids function as key promoters in the angiogenesis, invasion, migration and metastasis of OSCC, which is a research focus all along. In recent years, the effects of exosomes on other oral diseases such as periodontitis and oral lichen planus are also receiving attentions, providing us with a more comprehensive understanding about the roles that exosomes play in oral diseases. Moreover, exosomes containing multiple biological molecules display great potential to be exploited to assist clinical diagnosis and evaluate prognosis. Exosomes-based therapies are promising strategies in oral tissue regeneration, cancer treatment, and as drug delivery vehicles in the coming future. However, researches are still largely limited at present. Future studies should not only investigate the biological functions and precise molecular mechanisms of exosomes in oral diseases, but also address the clinical applications of exosomes to facilitate the clinical translation.

## Data Availability

Not applicable.

## Abbreviations

ASCs: Adipose-derived stem cells
CAFs: Carcinoma associated-fibroblasts
DP-MSCs: Dental pulp stem cells
EBV: Epstein–Barr virus
ESCRT: Endosomal sorting complex required for transport
ESHFMD: Extremely severe Hand, foot, and mouth disease
HFMD: Hand, foot, and mouth disease
IBD: Inflammatory bowel disease
ILVs: Intraluminal vesicles
LPS: Lipopolysaccharide
MDR: Multiple drug resistance
MSCs: Mesenchymal stem cells
BM-MSCs: Bone marrow-derived MSCs
MVBs: Multivesicular bodies
OLP: Oral lichen planus
OPMD: Oral premalignant disorders
OMECs: Oral mucosal epithelial cells
OSCC: Oral squamous cell carcinoma
OSCC-Exos: OSCC cell-derived exosomes
OLK: Oral leukoplakia
PDLFs: Periodontal ligament fibroblasts
PDLSCs: Periodontal ligament stem cells
pSS: Primary Sjögren’s syndrome
SGECs: Salivary gland epithelial cells
SHEDs: Stem cells derived from dental pulp of human exfoliated deciduous teeth
SIRPα: Signal regulatory protein α
TAM: Tumor-associated macrophages
